# Variation and inheritance of iron reductase activity in the roots of common bean (*Phaseolus vulgaris *L.) and association with seed iron accumulation QTL

**DOI:** 10.1186/1471-2229-10-215

**Published:** 2010-10-05

**Authors:** Matthew W Blair, Sharon JB Knewtson, Carolina Astudillo, Chee-Ming Li, Andrea C Fernandez, Michael A Grusak

**Affiliations:** 1 Biotechnology Unit and Bean Program, International Center for Tropical Agriculture (CIAT), Cali, Colombia; 2 Department of Pediatrics, USDA-ARS Children's Nutrition Research Center, Baylor College of Medicine, Houston, Texas, USA

## Abstract

**Background:**

Iron deficiency anemia is a global problem which often affects women and children of developing countries. Strategy I plants, such as common bean (*Phaseolus vulgaris *L.) take up iron through a process that involves an iron reduction mechanism in their roots; this reduction is required to convert ferric iron to ferrous iron. Root absorbed iron is critical for the iron nutrition of the plant, and for the delivery of iron to the shoot and ultimately the seeds. The objectives of this study were to determine the variability and inheritance for iron reductase activity in a range of genotypes and in a low × high seed iron cross (DOR364 × G19833), to identify quantitative trait loci (QTL) for this trait, and to assess possible associations with seed iron levels.

**Results:**

The experiments were carried out with hydroponically grown plants provided different amounts of iron varying between 0 and 20 μM Fe(III)-EDDHA. The parents, DOR364 and G19833, plus 13 other cultivated or wild beans, were found to differ in iron reductase activity. Based on these initial experiments, two growth conditions (iron limited and iron sufficient) were selected as treatments for evaluating the DOR364 × G19833 recombinant inbred lines. A single major QTL was found for iron reductase activity under iron-limited conditions (1 μM Fe) on linkage group b02 and another major QTL was found under iron sufficient conditions (15 μM Fe) on linkage group b11. Associations between the b11 QTL were found with several QTL for seed iron.

**Conclusions:**

Genes conditioning iron reductase activity in iron sufficient bean plants appear to be associated with genes contributing to seed iron accumulation. Markers for bean iron reductase (FRO) homologues were found with *in silico *mapping based on common bean synteny with soybean and *Medicago truncatula *on b06 and b07; however, neither locus aligned with the QTL for iron reductase activity. In summary, the QTL for iron reductase activity under iron limited conditions may be useful in environments where beans are grown in alkaline soils, while the QTL for iron reductase under sufficiency conditions may be useful for selecting for enhanced seed nutritional quality.

## Background

Iron deficiency anemia (IDA) is among the most common nutritional problems of human populations throughout the world, affecting more than 2 billion people to varying degrees [1]. While the deficiency is widespread, the lack of iron in the diet or inability to assimilate iron in sufficient quantities is most serious for children, adolescents, and women of child-bearing age [2,3]. Iron is an essential element for human growth and development that is needed for the Krebs cycle, for cytochrome function and for cellular respiration [4]. Most of the iron in the human body is part of hemoglobin, myoglobin, ferritin or transferrin [5].

Various strategies are used to combat IDA [1]. For example, supplementation with iron can be practiced at health centers through a liquid or injectable medicine. Drawbacks are the taste of the liquid medicine, teeth staining and need for outreach and a delivery mechanism [4]. Fortification is another approach for delivering iron to IDA susceptible populations that is best done with flours produced from cereals but tends to be costly and is not an option for whole grains, like legumes. An alternative to both of these is to increase the concentration of iron in diets through biofortification of staple foods [6]. Legumes are especially useful sources of micronutrients such as iron and have added advantage of high proteins. One important legume, common bean, has been targeted in the worldwide effort on biofortification as a strategic crop for increasing dietary iron for human beings.

Common bean is a major food staple of Eastern and Southern Africa and Latin America and with a total production of c. 25 M tons is the most widely grown food legume around the world, being highly valued in international trade and in regional markets [7]. Common bean like other legumes is known as the 'meat of the poor' due to their role as an economical alternative in the diets of people who cannot afford animal products. Varieties of common bean grain can be classified into two major genepools based on seed size differences, with Andean beans being large seeded and Mesoamerican beans being small seeded. The genepools differ in many respects including some basic physiological properties as well as nutritional characteristics and DNA polymorphisms. A range of results have shown that Andean beans tend to have higher seed iron concentration than Mesoamerican beans [8-10].

Iron reductases are members of the protein super-family of flavocytochromes and function in roots to convert iron from a plant unavailable form (ferric, Fe^3+^) to an available form (ferrous, Fe^2+^) that can be readily absorbed [11]. An iron reductase protein (FRO) is located within the plasma membrane, especially in root epidermal cells, where it is required for iron reduction [12,13]. Iron reductase activity is known to vary with plant growth conditions (e.g., soil pH and available iron concentration) and mutants in pea have been useful for analyzing the role of iron reduction in root iron acquisition [14]. Combined with root iron transporters and perhaps the release of phenolics, FRO is essential for iron uptake in Strategy I plants that do not produce siderophores for iron capture from soil [15,16].

The objective of this study was to evaluate iron reductase activity in common bean roots and to analyze the possible role of root iron reduction in the accumulation of seed iron. We did this by evaluating diverse germplasm grown at different iron concentrations for root iron reductase activity and then mapping quantitative trait loci for that activity in an inter-genepool cross population (DOR364 × G19833) that has been used for genetic mapping of seed iron concentration [17]. In addition, we identified two genetic markers for bean iron reductases and performed *in silico *mapping of a putative iron reductase gene (*Pv*FRO) by synteny analysis between soybean, Medicago and bean.

The analysis of iron reductase activity in common bean is part of a broader program of nutritional genomics being conducted within a biofortification program for common bean. Genomics in common bean benefits from a small genome of approximately 650 Mb (n = 11) and substantial synteny with other legumes which have been fully characterized for expressed genes (ESTs) or which have sequenced genomes [18]. Synteny analysis within the legumes has been conducted by various authors [19,20] and the common bean genome is known to be a simple diploid model of the soybean genome [21]. Nutritional genomics was first described as the analysis and modification of genes involved in the pathways leading to nutrient accumulation [22,23]. The analysis of iron reductase activity, aside from potentially influencing nutritional quality, is also important for adaptation of common beans to iron deficient alkaline or calcareous soils [24].

## Results

### Parental differences and germplasm diversity

The parents of the DOR364 × G19833 mapping population based on a high seed iron × low seed iron cross as described by Blair et al. [17] were evaluated in an initial randomized complete block experiment in the growth chamber with four replications to evaluate iron reductase activity across a range of iron concentrations that included 0, 1, 2, 5, 10 and 20 μM Fe(III)-EDDHA (Fe). Similarly, a set of 13 wild and cultivated genotypes were evaluated in the same conditions but with only two iron concentrations, namely at limited iron (2 μM Fe) and iron sufficiency (15 μM Fe). The mapping population was then evaluated in additional randomized complete block experiments with the same conditions but with 1 μM Fe used for the iron-limited treatment and 15 μM Fe for the sufficiency treatment based on the results from the parental screening.

**Figure 1:**
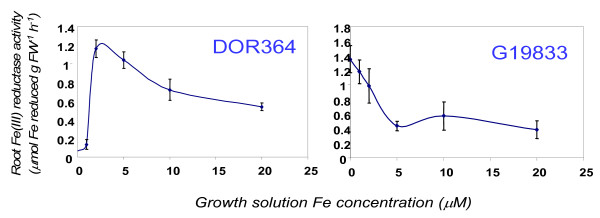
**Iron reductase activities of the parents of the DOR364 × G19833 population for plants grown for 12 days at various levels of hydroponic iron concentration**. Rates of iron reduction were expressed in μmol Fe reduced g FW^-1 ^h^-1^.

The first iron reductase assay identified interesting differences between the parents of the mapping population for their ability to reduce iron when grown at various hydroponic iron concentrations ranging from 0 to 20 μM Fe (Figure 1). These differences were more evident in plants grown at low Fe concentrations (iron limiting conditions) than at high iron concentrations (sufficiency conditions), such that G19833 had its highest iron reductase activity when grown at 0 μM Fe, while DOR364 exhibited very low iron reductase activity when grown at 0 or 1 μM Fe, but achieved higher rates when grown at 2 μM Fe. At 5, 10 and 20 μM Fe, the iron reductase activity of DOR364 was slightly higher than that of G19833, but the differences were not significant.

To evaluate whether these results were typical of other common bean germplasm, we selected a range of nine cultivated landraces and four wild accessions from the CIAT genebank collection (Table 1) to determine the variability in iron reductase activity when plants were challenged with iron-limited (2 μM Fe) and iron sufficient (15 μM Fe) growth conditions. The genotypes selected for this experiment were based on their known seed iron concentrations from the evaluation of the CIAT cultivated and wild core collections [8, CIAT unpublished). Among the cultivated genotypes, G11350 was selected for being high for seed iron within the Mesoamerican (small-seeded) genepool, while G11360 was selected for being low in iron within this genepool. Two other Mesoamerican beans were also evaluated, as was one intermediate type between the Andean and Mesoamerican genepools.

**Table 1:** Evaluation of diverse common bean germplasm for iron reductase activity (μmol Fe reduced/g FW/h) in 12-d-old plants grown under iron-limited (2 μM Fe) or iron-sufficient (15 μM Fe) conditions, and for seed iron concentration in mature, soil-grown plants.

Genotype	Genepool^1^	Seed Colour	Seed Fe Level^2^	Iron reductase activity in 2 µM Fe-grown plants	Iron reductase activity in 15 µM Fe-grown plants
					

**Cultivated**					

G11350	M	Small red	High (64)	0.835	0.350

G11360	M	Medium purple	Low (43)	0.454	0.165

G19227A	M	Small black	High (65)	0.879	0.047

G19833	A	Large yellow mottled	High (70)	1.054	0.123

G19839	A	Large yellow mottled	High (59)	1.083	0.200

G21078	A	Large cream	Low (36)	0.049	0.307

G21212	M	Small black	ND	1.089	0.482

G21242	A	Large cream mottled	High (90)	0.713	0.528

G21657	A-M	Medium cream mottled	High (85)	0.639	0.231

					

**Wild**					

G23585	A	Very small grey speckled	ND	0.048	0.036

G24390	M	Very small grey speckled	ND	0.187	0.036

G24404	C	Very small grey speckled	ND	1.039	0.705

G24423	C	Very small grey speckled	ND	0.180	0.342

1/*Genepools: A = Andean wild or cultivated, C = Colombian wild, M = Mesoamerican wild or cultivated.

2/Iron level in parts per million (ppm or mg/kg) from Islam et al. (2002) and CIAT unpublished (iron level given in parentheses [ppm; dry wieght basis]; high = above 55 ppm, low = below 55 ppm, with 55 ppm being the average iron concentration for the crop), ND = not determined.

Within the Andean (large-seeded) genepool, G19833, G19839 and G21242 were selected for being high in iron while G21078 was selected for being low in iron concentration. The iron reductase activity was indeed higher in the genotypes with high seed iron than those with low seed iron, especially for G21078 or G11360. The correlation within the cultivated genepools between seed iron concentration and iron reductase activity at 2 μM Fe and at 15 μM Fe were r = 0.575 and r = 0.327, respectively, with neither being significant. Our interest in correlating iron reductase activity with seed iron concentration was part of our long term goal of evaluating whether iron reductase activity was potentially predictive of the eventual accumulation of iron in that genotype's seeds.

Iron reductase activity differences were not evident between the cultivated genotypes from the Andean versus the Mesoamerican genepool based on t-tests at either growth iron concentration level, but this might have been due to the selection of genotypes for contrasting seed iron concentrations. There was some suggestion that Andean beans had slightly higher iron reductase activity when grown at 2 μM Fe and at 15 μM Fe, if we exclude G21078, the low iron Andean bean. G21212 a Mesoamerican small black bean had surprisingly high levels of iron reductase activity under both conditions. G19277A, the other small black bean, had high iron reductase activity under iron-limited conditions but not under sufficiency conditions. G21657, the inter-genepool type, was intermediate.

Among the wild genotypes, which were very much smaller seeded than any of either the Mesoamerican or Andean common beans, iron reductase activity was notably low in plants grown at 2 μM Fe compared to the cultivated beans. One exception was G24404, which had high iron reductase activity comparable to the high seed iron Andean bean genotypes. This genotype along with G24423 also had high and moderate iron reductase activity in 15 μM Fe grown plants, while the other wild beans had very low activity at both growth levels.

It was interesting that wild beans from both the Andean and Mesoamerican genepools behaved in a similar fashion at either level of iron, while the genotypes from the Colombian wild genepool (a different non-domesticated genepool) were those that had high to intermediate iron reductase activity depending on the level of iron provided.

### Population distribution and QTL detection

**Figure 2:**
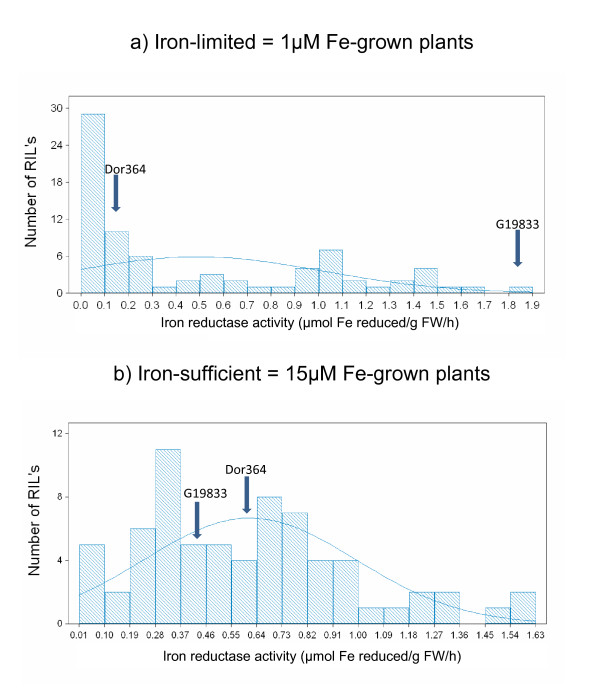
**Population distributions for iron reductase activity (μmol Fe reduced/g FW/h) under a) iron-limited (1 μM) and b) iron-sufficient (15 μM) growth conditions in the DOR364 × G19833 recombinant inbred line mapping population**. Maternal (DOR364) and paternal (G19833) root iron reductase values indicated by arrows.

In the experiment using the DOR364 × G19833 recombinant inbred lines, significant differences were found for iron reductase activity between the population distributions obtained with iron-limited (1 μM Fe) versus iron sufficient (15 μM Fe) plants, as shown in Figure 2. In this case, 1 μM Fe was selected as the level of iron to test the population based on the best contrast between the parental genotypes in the initial parental screening experiment. The definite bimodal nature of the population distribution at 1 μM Fe and the somewhat bimodal population distribution at 15 μM Fe shows that inheritance of iron reductase activity was controlled by one or a few genes, respectively.

The range of iron reductase activity values obtained with iron-limited plants (0 to 1.9 μmol Fe reduced/g FW/hr) was slightly broader than with iron sufficient plants (0 to 1.7 μmol Fe reduced/g FW/hr). The averages at low and high iron growth concentrations were similar at 0.49 and 0.61 μmol Fe reduced/g FW/hr, respectively. The position of the parental values relative to the lines showed there to be transgressive segregation at the higher iron concentration where DOR364 and G19833 were similar in iron reductase activity but the RILs were much more widely distributed in their reductase activity. Transgressive segregation was less evident at the lower iron concentration where DOR364 was similar to one set of RILs that had low iron reductase activity and G19833 to the other set having high iron reductase activity in the bimodal distribution observed for 1 μM Fe-grown plants.

**Figure 3:**
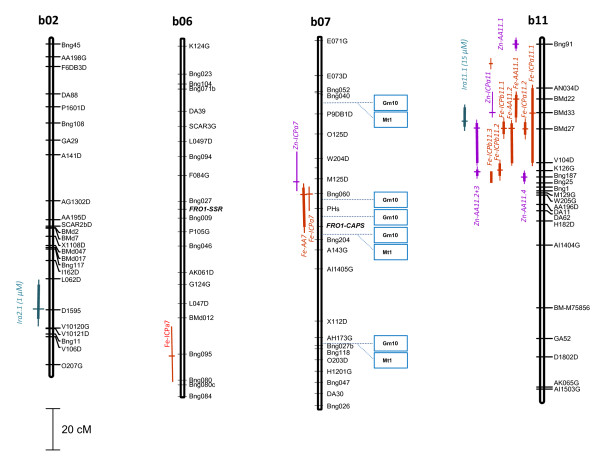
**Location of iron reductase (FRO-SSR and FRO-CAPS) gene markers and quantititative trait loci (QTL) for iron reductase activity (*Ira2.1 and Ira11.1*) on common bean linkage groups b02, b06, b07 and b11**. Vertical lines for each QTL represent the range of the QTL that are above the LOD threshold; horizontal marks on the lines indicate the LOD peak for the QTL. QTL for seed iron and zinc concentrations using ICP or AAS measurements as per Blair et al. (2009a). Synteny results for linkage group b07 with *Medicago *(Mt) and soybean (Gm) chromosomes as per Galeano et al. (2009) in the region of the FRO1-CAPS marker.

QTL analysis using composite interval mapping confirmed that there was one major QTL under iron sufficiency (15 μM Fe) and this QTL was on a different chromosome from the one under iron-limited growth (1 μM Fe) as shown in Figure 3. Therefore, it can be postulated that iron reductase activity was conditioned by more than one locus, with one iron reductase-related locus on Chromosome b02 contributing to the trait in iron-limited plants and another iron reductase-related locus on Chromosome b11 contributing to the trait in iron-sufficient plants. The QTL detected under iron-limited growth had a higher LOD score (5.94) than the one under sufficiency (2.96) and correspondingly the R^2 ^values were higher for the first QTL than the second (0.268 and 0.162, respectively). TR^**2 **^values were 0.473 and 0.361, respectively, for the two QTL. Alleles increasing iron reductase activity detected at both of these QTLs were contributed by the parent G19833. To further understand the relationship of iron reductase and seed iron we compared the QTL for iron reductase activity and QTL for seed iron concentration from Blair et al. [17]. An overlap of QTL controlling seed iron accumulation in the DOR364 × G19833 population with QTL for iron reductase activity was found on chromosome b11, but not for the other iron reductase activity QTL on chromosome b02.

### Genetic mapping and synteny analysis

Our first goal in this section of our research was to develop a DNA marker for a common bean iron reductase gene (*Pv*FRO) based on EST searches and cloning of orthologs of the iron reductase gene, while our second goal was to use *in silico *synteny mapping to discover iron reductase genes in the soybean genome and in equivalent common bean loci based on the comparative mapping described in Galeano et al. [21].

For our FRO marker development, the first SCAR marker we used from *P. sativum *did not amplify as expected in common bean and was not used further. A second marker we developed based on an SSR in the NAD-binding domain of a FRO homologue produced a single band on polyacrylamide gels of 235 bp for DOR364 and 220 bp for G19833, which was mapped to the middle of chromosome b06 within 5 cM of the flanking RFLP markers Bng009 and Bng027. The third primer pair tested based also on this region of the gene produced a single sized PCR product of 790 bp on agarose gels which when digested with *Hind*III showed a polymorphism between the parents (G19833 band remaining undigested compared to DOR364 band digested to 750 and 40 bp fragments) which was used to genetically map the marker to the middle of chromosome b07 at a distance of 9.8 cM from the phaseolin gene.

Figure 3 shows the location of the new markers, their association with seed iron QTL from Blair et al. [17] and the locations of the QTL for iron reductase activity. It was notable that the iron reductase activity QTL were not associated with the FRO markers but did overlap with a cluster of seed iron and zinc QTL on linkage group b11 from Blair et al. [17], that were near the markers BMd22 and BMd33. In addition, the FRO-CAPS-*Hind*III marker was associated with two seed iron QTL on linkage group b07 from that same study; however, the FRO-SSR on linkage group b06 was not associated with any previous seed iron QTL or with the ICP data. Table 2 summarizes the results of single point analysis with each of the new and flanking markers.

**Table 2:** Association of FRO loci and flanking markers with iron reductase activity (IRA) under iron limited (1 μM Fe) and iron sufficient (15 μM Fe) growth conditions and with seed iron concentration.

Locus^1^	LG^2^	IRA (1 uM)		IRA (15 uM)		Fe-ICP^3^		Fe-AAS^3^	
		
		LOD	R2	LOD	R2	LOD	R2	LOD	R2
									

FRO - SSR	6	0.33	.012	0.04	.002	1.23	.032	0.10	.004

Bng009	6	0.39	.014	0.05	.047	0.95	.026	0.11	.006

Bng027	6	0.02	.005	1.08	.002	1.15	.029	0.21	.004

									

FRO - CAPS (*Hind*III)	7	0.97	.017	0.05	.029	0.06	.002	2.31	.062

Bng060	7	0.14	.004	0.63	.027	1.02	.010	3.85*	.105*

Phs	7	0.12	.004	0.23	.018	1.46	.015	3.30*	.106*

1/The significance of successfully mapped FRO loci and their flanking markers are indicated in terms of LOD scores and R^2 ^values for iron reductase activity (IRA) and

2/LG (Linkage group) numbering according to Blair et al. (2003)

3/.Fe-ICP and Fe-AAS as described in materials and methods and Blair et al. (2009a), respectively.

* indicates significance at LOD threshold of 2.5 in single point regression analysis.

**Figure 4:**
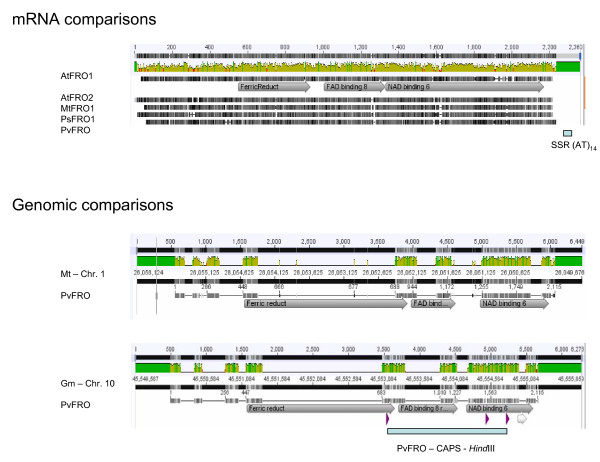
**Gene model and homology of common bean iron reductase gene (*Pv*FRO) with other iron reductase genes from arabidopsis (*At*FRO) or pea (*Ps*FRO) followed by synteny analysis with *Medicago *and soybean chromosomes**. Pfam annotation used with ferric reductase, FAD8 and NAD6 binding as major domains. Upper scale indicates nucleotide number in both the mRNA and genomic comparisons along with levels of homology. Lower horizontal bars represent each gene with intron/exon structure shown for *Pv*FRO. SSR marker from 3' UTR indicated below gene model and CAPS marker with *Hind*III site indicated below genomic comparisons with soybean.

For the synteny analysis, the FRO sequence from common bean linked to chromosome b07 (GenBank accession, HM440564) was found to be homologous to Pfam entries for FRO1 and FRO2 from *A. thaliana*, FRO1 from *M. truncatula *and FRO1 from *P. sativum*, the initial starting point for discovery of the FRO orthologs in *P. vulgaris*. All the genes were found to have the ferric reductase domain toward the middle of the coding sequence followed by FAD and NAD binding domains at the 3' end of the gene. Comparisons of these sequences to genomic sequences of soybean and medicago found that the gene model for the *Pv*FRO on chromosome b07 consisted of 8 exons and 7 introns in both cases as seen in Figure 4 where the position of the CAPS marker is also shown.

Macro-synteny analysis based on Galeano et al. [21] for both legumes placed the FRO gene in the middle of chromosome b07 of common bean exactly where the CAPS marker mapped. The homologous loci in the other legumes were 1) near the end of chromosome Gm10 of soybean at 45 Mb (mega-bases) or approximately 23 Mb past the centromere with no homeologous position elsewhere in the soybean genome; and 2) at a locus 26 Mb into chromosome 1 of Medicago which being diploid was not expected to have a duplicate position. It was interesting that the position of introns was conserved with the 4th being the largest and interrupting part of the ferric reductase domain. Several ESTs from common bean were found with homology to parts of the NAD or FAD domains but no other ESTs have been found covering the iron reductase domain.

## Discussion

For all the common bean genotypes investigated in this study, iron reductase activity was elevated in response to iron-limited growth conditions. This was especially evident with DOR364, G19833, and other genotypes, where at low iron concentrations (1 μM Fe but not 0 μM Fe) root iron reduction was 2.5 times the activity under higher iron concentrations (10 μM Fe and above). This has been observed before in peas and other legumes, where low iron supply is known to induce increases in iron reductase activity [25,26]. In some genotypes such as DOR364, iron reductase activity at 0 μM Fe might be reduced due to a need for iron in the enzyme itself. Iron reductase activity is known to be the rate-limiting enzyme for iron uptake since iron transporters, which are the other important element of root iron acquisition, do not reach saturation at normally achieved concentrations of ferrous iron [27]. Initial differences in the parents showed that DOR364 and G19833 had different sensitivities to growth on low iron concentration, with G19833 having much higher iron reductase activity at 1 μM Fe, the lower level selected for the RIL study. Apart from the intensively studied parental genotypes, wide variability was noted for iron reductase activity among the cultivated and wild common beans. Among the cultivated beans, one notable conclusion was that high seed iron accumulators generally had higher iron reductase activity than low seed iron accumulators within each genepool, suggesting a link between root uptake and seed loading of iron in common bean contrasting with results with an iron reductase mutant in pea [28]. Andean beans had higher iron reductase activity especially at low iron growth conditions and are generally higher for seed iron as well [8-10] both of these results having implications for their use in biofortification or adaptation to low iron soils [12,29].

While among the cultivated Andean genepool there was high iron reductase activity under iron-limited growth, there was lower activity under sufficiency conditions, while the cultivars from the Mesoamerican genepool had moderate to low activity under the two growth conditions. Genotypes that accumulated high seed iron concentrations, such as G11350 (Mesoamerican), G19833, G19839 and G21242 (all Andean), generally had high iron reductase activity under iron-limited growth and moderate activity under iron sufficiency; in other words, they had higher and apparently more consistent iron reductase activity than those genotypes that did not accumulate high seed iron especially among Mesoamerican genotypes. Notably, one Andean genotype, G21078, which was low in seed iron concentration, had very low iron reductase activity in iron limited and iron sufficient growth conditions. Small-seeded black beans may have an adaptation to low iron soils in some cases as both G19277A and G21212 were high in iron reductase activity under both growth conditions.

Differences in seed iron levels and adaptation to low iron available soils have been found in common bean cultivars [8-10,30,31] and may have been selected for during domestication and development of common bean races [32]. Compared to pea, peanut and soybean, the only other legumes studied for this trait [29,33,34], iron reductase activity responses were similar in roots of iron limited common beans and these results agree with those of Bienfait et al. [24]. Iron deficiency is a problem of high pH soils in certain bean temperate or dryland production regions but is not generally a problem in tropical countries with low pH soils.

Wild beans which have never been studied before for this trait, appeared to be especially low in iron reductase activity, although one genotype was high in iron reduction. It is typical for greater variability to be present in wild beans compared to domesticated beans as a bottleneck in genetic diversity was suggested to have occurred at domestication [35] and wild beans can be a source of high seed minerals [36,37]. Natural selection on alkaline soils may have allowed the development of some populations of wild beans with high iron reductase activity and this may have been the case for the Colombian wild bean G24404 which has been analyzed for its diverse phenology compared to Andean beans by Blair et al. [38].

The differences in iron reductase activity between parents allowed us to analyze the inheritance in the DOR364 × G19833 population. Iron reductase activity under deficiency conditions appeared to be a monogenic trait controlled by a single QTL (*Ira*2.1) mapping to chromosome b02. Meanwhile, the inheritance at iron sufficient conditions was slightly more complex, although a single QTL on chromosome b11 (*Ira*11.1) was still the most determining factor for iron reductase activity. This QTL was very interesting because it aligned with QTL for seed iron accumulation from Blair et al. [17]. Although the correlation of ICP seed iron accumulation with iron reductase activity in this study was not significant at either 1 μM (r = 0.021) or at 15 μM (r = -0.159), it was interesting to see that the iron reductase activity QTL was located in the same place as a seed iron concentration QTL for chromosome b11.

The possibility of an association of iron reductase activity with more iron in the seed is of interest to biotechnologists [23,39]. This is the first study of iron reductase QTL as a component of iron homeostasis, although seed iron concentration has been measured in a range of QTL studies in arabidopsis [40,41], common beans [17,42] and other legumes [43,44], just to mention those of interest here. Mutant analysis has been of great utility for peas in the group of Grusak and collaborators to study aspects of iron reductase activity [25], iron transporters [27,45], root changes during iron deficiency [46], seed loading of iron [47] and other aspects of iron homeostasis; results which suggest that mutagenesis may be an interesting method of dissecting iron uptake in common bean as well as suggested by Blair et al. [48].

Surprisingly, neither of the QTL identified in this study for iron reductase activity aligned with the positions of the markers for *Pv*FRO orthologs that were successfully mapped on chromosomes b06 and b07. This may have been expected, since in other research the control of iron reductase activity has been postulated to be under the control of a shoot to root signal that induces reductase expression [49]. It was not surprising that we found two map locations for the FRO orthologs since iron reductases are known to be an eight-member gene family in *Arabidopsis thaliana*, where paralogs exist and some genes have different functionality and expression patterns in roots versus several diverse above ground organs [50]. Although the family has not been characterized in soybean we found one homologous copy of FRO on chromosome Gm10 with the equivalent positions in common bean being on chromosome b07 where the CAPS marker mapped to. Medicago synteny confirmed this position.

Given that the QTL for iron reductase activity do not map together with the loci for the FRO genes, either as mapped markers or in synteny analysis, we may conclude that some other gene is controlling iron reductase activity. This could be due to another copy of the FRO gene [50] or an iron homeostasis regulator [51]. In the case of the QTL found under iron-limited growth, this may be equivalent to the *dgl *(degenerative leaves) or *brz *(bronze leaves) loci in pea since both are involved in the constitutive up-regulation of iron reductase activity [25,49]. However, the state of synteny mapping between pea and other legumes make it difficult to know if the mutants or even the FRO homologues are in equivalent positions between common bean and pea [52].

From a physiological standpoint, any gene or signal stimulating iron reductase activity would be an important component needed to match root iron uptake with the above ground plant needs [49] and might be a candidate for the QTL we identified. For example, the gene for such a signal could be related to ethylene induced local changes in root cell restructuring which affect iron reduction and transport [46], to a shoot to root signal, or to a transcription factors affecting iron homeostasis. If this is the case, this signal may change in intensity throughout the life-cycle of the plant, such as during active seed-fill when iron is being loaded through the phloem to the developing seed [28].

## Conclusions

In our germplasm survey, broad diversity for iron reductase activity was found in common bean with some differentiation of the Andean and Mesoamerican genepools and substantial differences between cultivated and wild beans. Wild beans may not have the same requirement for iron uptake given their low seed yield and small leaves except for a few accessions from the Colombian genepool, while some Andean beans might have been selected for high iron reductase activity from being grown on higher pH/organic material soils which are low in iron concentration. Mesoamerican beans would be less likely to have high iron reductase activity since they are generally grown on acid soils with plenty of iron. As was found for other legumes, iron limited conditions were seen to induce iron reductase activity, while at higher iron levels this activity was repressed. The genotypic differences in iron reductase activity between DOR364 and G19833 allowed the discovery of differentially expressed QTL at iron limited and iron sufficient growth conditions in the mapping population. These QTL (*Ira*2.1 and *Ira*11.1) were independent and positioned on linkage groups b02 and b11, respectively. The second of these QTL was associated with seed iron accumulation QTL but was not associated with the *in silico *or genetic map position of FRO orthologs in common bean based on new markers and synteny mapping with soybean and medicago. The FRO locus on linkage group b07 may have had an effect on seed iron accumulation but the mechanism would be unknown. Meanwhile, the *Ira*11.1 QTL that clustered with seed iron QTL on b11 would be interesting for further dissection. All of these novel results represent achievements in the application of nutritional genomics to common bean biofortification.

## Methods

### Plant materials

An initial experiment was conducted on 14 common bean genotypes from the FAO designated collection held at CIAT including 10 cultivated and 4 wild genotypes. Two subsequent experiments involved a population of recombinant inbred lines (RILs) from the cross of DOR364 × G19833 as described in Blair et al. [53] and Beebe et al. [54] for one of the experiments, while the other experiment involved the parents of this population. The parents represented the Mesoamerican (small-seeded) and Andean (large-seeded) genepools, respectively. The RILs were all in the F11 generation and were produced along with the other genotypes by the International Center for Tropical Agriculture (CIAT) at the Darien site as described in Blair et al. [17].

### Plant growth and iron reductase activity measurements

For all studies, seeds were germinated for 4-6 days on germination paper and then planted in hydroponic media per methods described in Grusak et al. [25]. Plants were grown as sets of four plants per 4.5 L of nutrient solution; the solution contained 1.2 mM KNO_3_, 0.8 mM Ca(NO_3_)_2_, 0.3 mM NH_4_H_2_PO_4_, 0.2 mM MgSO_4_, 50 μM KCl, 12.5 μM H_3_BO_3_, 1 μM MnSO_4_, 1 μM ZnSO_4_, 0.5 μM CuSO_4_, 0.1 μM H_2_MoO_4_, 0.1 μM NiSO_4_, 1 mM MES buffer (2,4-morpholino-ethane sulfonic acid), adjusted to pH 5.5, and various concentrations of Fe(III)EDDHA [ethylenediamine-N, N'bis(o-hydroxyphenyl) acetic acid]. Plants were maintained in hydroponics for 12 days; nutrient solutions were changed 7 and 10 days after planting. All plants were grown within environmentally controlled growth chambers (PG2V; Controlled Environments Inc., Pembina, ND, USA) using a mixture of fluorescent and incandescent lamps that provided a photon flux density of 350 μmol m^-2 ^s^-1^. Growth chamber settings were 15 h light at 22.5°C, 9 h dark at 17.5°C, and a relative humidity of 50 + 5%.

Iron reductase activity was measured with excised, entire root systems, using the 12-day old plants, according to the methods described in Grusak et al. [25]. Assays were run under low light conditions for 40 min using 100 μM Fe(III)EDTA (ethylenediamine tetraacetic acid) as the ferric iron source and 100 μM BPDS (bathophenanthroline disulfonic acid) as the ferrous iron chelator. Roots were gently blotted immediately after the assays and weighed to determine fresh weight. Absorbance values for aliquots of the assay solution were obtained spectrophotometrically at 535 nm to determine concentrations of Fe(II)BPDS_3 _(generated in the assay); an aliquot of the solution that had no roots during the assay was used as blank. Rates of iron reduction (μmol Fe reduced g FW^-1 ^h^-1^) were determined using a molar extinction coefficient of 22.14 mM^-1 ^cm^-1^. Values presented in the paper, or used in QTL determinations, were the average values derived from a minimum of four root systems.

### Marker development for iron reductase

Several techniques were used for marker development. First, a sequence characterized amplified region (SCAR) marker for the FRO1 gene from *P. sativum *(GenBank accession AF405422) was used in common bean [52,55]. Following that, a search was made of FRO-like sequences from the BEST project http://lgm.esalq.usp.br/BEST database, with one hit (contig 449) containing a simple sequence repeat for which primers (Forward 5'-CCACAGCTTTGATCTCTA GC-3' and Reverse 5'-CACAGAAAACTGAGCATTCA-3') were designed with the software Primer 3.0 http://frodo.wi.mit.edu/primer3. Furthermore, primers for PvFRO (For-5'-GAGGCCTACGTTACCAGAGAAAA--3', Rev 5'-CGGTGTTGGAACTTCCACATTC-3') were designed from putative FRO mRNA sequence cloned by degenerate primers (GenBank accession, HM440564) and used in a cleaved amplified PCR sequence (CAPS) reaction using the enzyme *Hind*III. Both SCAR and CAPS markers were evaluated on 1.5% agarose gels run in 0.5X TBE; while the microsatellite marker was run on 4% polyacrylamide silver stained gels as described in Blair et al. [53]. Genetic mapping of successful markers was performed with Mapmaker v. 3.0 [56] and a minimum LOD of 3.0 and using the genetic map from Blair et al. [17].

### QTL analysis

QTL were detected first with composite interval mapping (CIM) analysis that was carried out using the software program QTL Cartographer v. 2.5 [57] and the genetic map for the DOR364 × G19833 used in Blair et al. [17] along with the markers for iron reductase genes described above. The following parameters were used for QTL detection: 10 cM window size, 1 cM walkspeed, 5 significant background markers, analysis by forward and backward multiple linear regression for each chromosomal position with a global significance level of 5% and probability thresholds of 0.05 for the partial F test for both marker inclusion or exclusion. In the CIM analysis, determination coefficients were calculated for each interval separately (R^**2**^) and for each interval given the background markers (TR^**2**^) to determine the phenotypic variance explained by a single QTL. QTL were reported for LOD > 2.5 and results were displayed using QTL Cartographer and represented graphically with standard drawing software, to designate genomic regions that proved to be significant in the analysis described above. Single point regression analysis was conducted for the successful FRO markers.

### Synteny analysis

The synteny analysis was as described in Galeano et al. [21] using the soybean (*Glycine max *L.) and medicago (*Medicago truncatula *L.) chromosome sequences from Phytozome and from the Legume Information System, respectively, and a search for ESTs from the common bean unigene set based on similarity to the *P. sativum *FRO1 gene. Genious v. 8 software was used to align cDNA sequences from common bean against sequences of pfam entries for *At*FRO1 and 2 from *Arabidopsis thaliana*, as well as *Mt*FRO1 and *Ps*FRO1 from medicago and pea, respectively, and also against chromosomal sequences of soybean and medicago, the two legumes which have genome sequence data. In addition, the EST assembly from Galeano et al. [21] was used to find additional contigs similar to *Ps*FRO1 based on the full set of ESTs from common bean assembled with EGassembler.

## Authors' contributions

MWB and MAG planned the study and obtained funding for the research. SJBK carried out iron reductase assays, CA mapped the markers and developed SSR primers for FRO1 homologs, CML cloned and developed markers for PvFRO, while CA and ACF carried out the QTL and synteny analyses, respectively. MWB wrote the paper with contributions from CA and MAG. All authors read and approved the final manuscript.
